# Integrated geological datasets for the Zandrivierspoort Formation banded iron formation, Rhenosterkoppies Greenstone Belt, South Africa

**DOI:** 10.1016/j.dib.2026.112831

**Published:** 2026-05-07

**Authors:** Lowanika Victor Tibane, David Mamba, Zakhele Handsome Nkosi

**Affiliations:** a Department of Geosciences, Nelson Mandela University, South Campus, Port Elizabeth, 6031, South Africa; b The Confidence Reef 631, Heiderkruin, Roodepoort, 1724, South Africa; c Department of Geology, University of Pretoria, Hatfield Campus, Pretoria, 0001, South Africa

**Keywords:** Zandrivierspoort Formation BIF, Least-altered BIFs, Detrital influence, Hydrothermal input, Physiochemical setting

## Abstract

Banded iron formation (BIF) deposits occur within the Rhenosterkoppies Greenstone Belt, situated along the northern margin of the Kaapvaal Craton in Limpopo Province, South Africa. A total of 20 representative BIF samples were collected, comprising ten outcrop samples from exposed BIF units and ten core samples selected from borehole ZDRP and ZDRT. The core samples represent sections where 3 to 25 m thick BIF units were intersected at depths reaching up to 200 m. Petrographic analysis of 20 thin sections, examined under transmitted and reflected light microscopy, provides detailed lithological, mineralogical, textural, and deformational data. Texturally, the BIFs are fine- to medium-grained, showing dark grey to black when fresh but reddish-brown when weathered. Mineralogically, the BIF units comprise magnetite (∼40%), quartz (∼40%), hematite (∼15%) and minor ∼5% contribution from goethite, muscovite, amphibole, and sulfide minerals, as confirmed by X-ray diffraction (XRD) data. Major elements data measured by X-ray fluorescence (XRF) are dominated by SiO_2_ (37.49 to 66.49 wt.%) and FeO (25.01 to 42.55 wt.%). Trace and rare earth element data, obtained using laser ablation inductively coupled plasma mass spectrometry (LA-ICP-MS), range from 6.77 to 140.95 ppm. These elements display a negative correlation with Fe yet a positive correlation with SiO_2_, accompanied by a weak positive Eu anomaly and a slightly negative Ce anomaly. Collectively, these data provide a basis for understanding Precambrian BIF systems, their geological evolution, and economic potential.

## Graphical abstract

**Figure:**
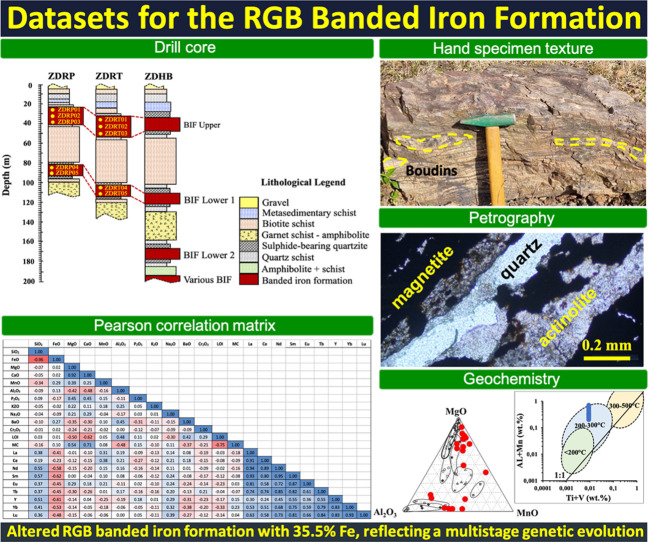

Specifications Table

**Table:** 

Subject	Earth & Environmental Sciences
Specific subject area	Economic Geology
Type of data	Table, Image, Chart, Graph, Figure, Raw, Analyzed, Filtered, Processed
Data collection	Structural measurements were recorded using a Brunton-type silver compass equipped with a clinometer to determine declination and inclination. Geographic sampling locations were captured with a Global Positioning System (GPS) in degrees, minutes, and seconds (DMS). Zandrivierspoort boreholes ZDRT and ZDRP, drilled to depths of 115 m and 135 m, respectively, were geologically logged and photographed (Table 2). Twenty standard 30 µm thick, thin sections were prepared for petrographic analysis using transmitted and reflected light microscopy (Fig. 3). The mineralogical data were obtained using X-ray diffraction (XRD) method. Major element concentrations were determined via X-ray fluorescence (XRF) (Table 4), while rare earth element (REE) data were obtained using LA–ICP–MS (Table 5) [1]. REE values were normalized to Ivuna-type carbonaceous chondrites (CI) by multiplying by a factor of 1.36 following established methods [2,3]. Europium and cerium anomalies were calculated as Eu/Eu* = Eu_n_/(0.67Sm_n_ + 0.33Tb_n_) and Ce/Ce* = log[Ce_n_/(La_n_ x Nd_n_)^1/2^] [[4], [5], [6]].
Data source location	Department of Geosciences, Nelson Mandela University, Port Elizabeth, Eastern Cape Province, South Africa, 6031.
Data accessibility	Repository name: Mendeley Data Data identification number: doi: 10.17632/n9thzj2f8y.2 Direct URL to data: https://data.mendeley.com/datasets/n9thzj2f8y/2
Related research article	Submitted

## 1 Value of the Data

• This dataset provides field observations, thin section description, whole-rock geochemical measurements, and magnetic content.
• The petrographic data of representative BIF samples illustrate mineral assemblages, textures, and deformation features.
• Major and trace element data denote depositional environment, detrital input, and metamorphic modification of the RGB BIF.
• Textures and chemical signatures reflect the physicochemical conditions of RGB BIF iron mineralization and subsequent metamorphic overprinting.
• The mineralogy and magnetic data provide estimates of iron grades and evaluation of the potential economic value of the modified Zandrivierspoort Formation BIF.

## 2 Background

Banded iron formation (BIF) deposits occur within the Zandrivierspoort (ZD) Formation. Geologically, the ZD Formation forms part of the Rhenosterkoppies Greenstone Belt (RGB), which is a subdivision of the Petersburg Greenstone Belt (PGB), situated along the northern margin of the Kaapvaal Craton, South Africa [7]. The BIF units represent chemical sedimentary rocks characterized by alternating Fe-rich and Si-rich layers [8,9]. Economically viable iron ores are largely derived from the Precambrian sedimentary sequences. However, the sources of iron in the BIFs remains debated. Generally, BIF deposition occurred during the Archean to Paleoproterozoic (3.8–1.8 Ga) and the Neoproterozoic (∼0.8–0.6 Ga) [[7], [8], [9]]. The most extensive period of BIF deposition occurred around 2.5 Ga, prior to the Great Oxidation Event (2.4–2.2 Ga), followed by another significant BIF deposition phase at ∼1.9–1.8 Ga, and a later reappearance during the Neoproterozoic [7,8]. Numerous BIF genesis models suggest that Fe was supplied through the weathering of continental materials via fluvial input, aeolian dust, glacial meltwater, and coastal erosion [7,[9], [10], [11]]. In contrast, alternative models propose volcanogenic and hydrothermal sources, where Fe was introduced directly from submarine volcanic activity [12]. Nonetheless, BIF formation likely involved a combination of continental weathering and hydrothermal inputs [12]. According to literature [13], BIF deposits formed in deep marine environments and exhibit compositional variability, including sulfide-, oxide-, silicate-, and carbonate-rich types.

For the RGB BIFs, the proposed genetic models include structural, syn-genetic, compositional, hypogene, and supergene models [7]. This diversity of the proposed models reflects the complex interplay of depositional processes, tectonic impacts, and post-depositional metamorphic modifications that have influenced the formation and evolution of the iron-rich deposits.

The current dataset documents the lithological, mineralogical, structural features, and geochemical characteristics of BIF deposits of the ZD Formation in the RGB, by integrating geological field mapping, sampling of representative outcrop samples where BIF units are exposed and drill core samples where BIF layers were intersected by bore ZDRT and ZDPT. Petrographic data obtained from thin sections using transmitted and reflected light microscopy, geochemical data of major, trace, and rare earth elements were incorporated to record geological information of the relatively under-documented Precambrian BIF occurring within the RGB. The dataset is provided in a structured and accessible format, enabling data reusability for further geological studies.

## 3 Data Description

### 3.1 Location description

**Fig. 1:**
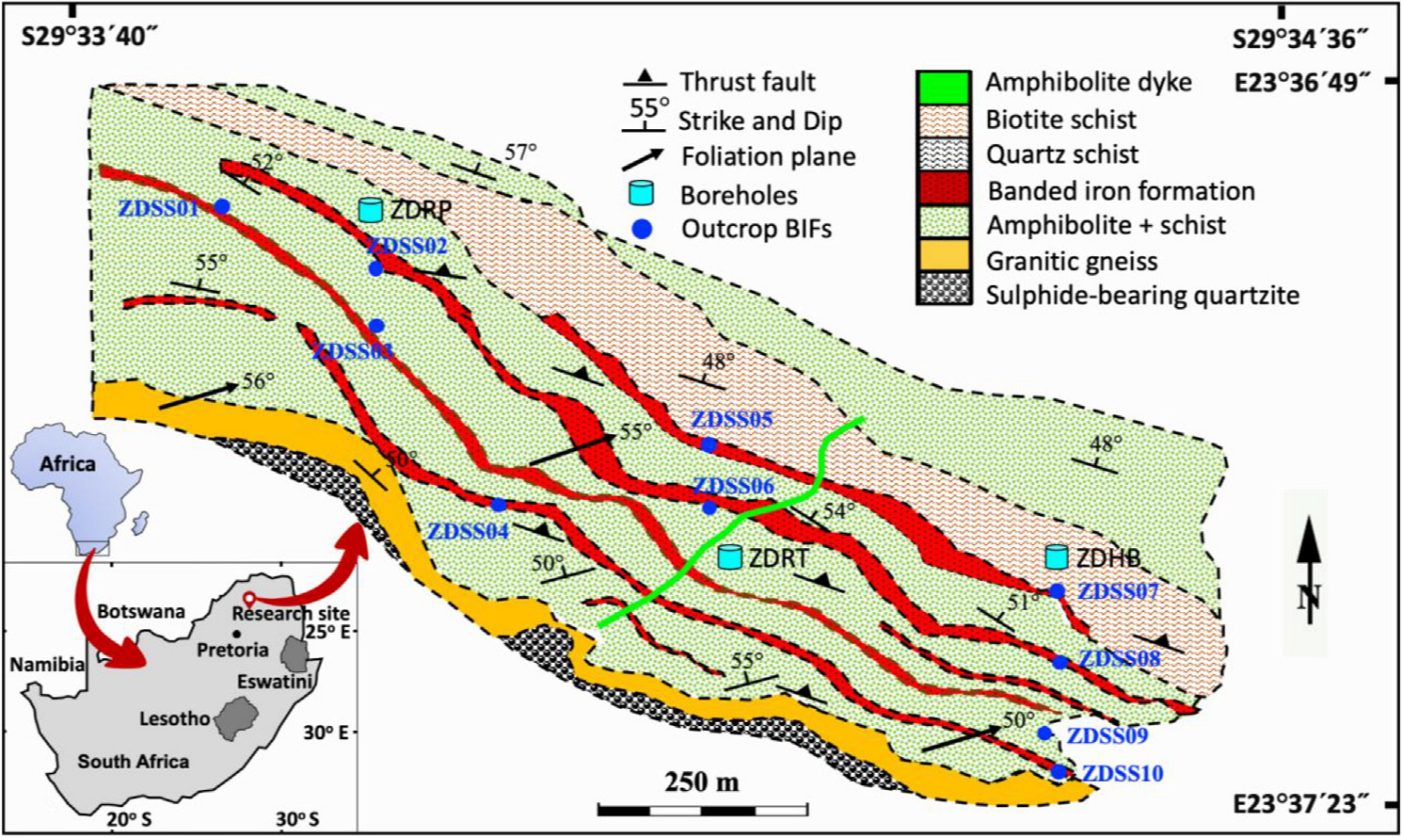
Location and geological map showing the distribution of lithologies, sampling sites and boreholes stations.

Geographically, the ZD Formation is located within the Molemole Local Municipality, roughly 35 km north of Polokwane in Limpopo Province, South Africa. This location is ∼200 km northeast of Pretoria and found at 23°37′17.21″S and 29°38′59.99″E (Fig. 1).

### 3.2 Sample description

A detailed description of the selected BIF samples from the ZD Formation is provided in Table 1 and includes sample identification numbers, stratigraphic positions, sampling depth intervals, and mineralogical characteristics for both ZDRP and ZDRT drill cores, as well as for representative outcrop lithologies. The exact geographic coordinates of individual boreholes are not disclosed due to company confidentiality, nevertheless their relative positions are indicated on the geological map (Fig. 1).

**Table 1:** Lithological descriptions of outcrop and borehole ZDRP and ZDRT BIF samples.

BIF Type	Sample, Depth (m)	Sample Description
**Altered laminated BIF**	ZDSS01 ZDSS03 ZDSS04 ZDSS05 ZDSS07 ZDSS08 ZDSS09 ZDSS10 ZDRP02 (33.20 m)	Altered laminated BIF samples are slightly to moderately weathered and display brownish to reddish coloration (Fig. 3c). The rocks are competent and difficult to break with a geological hammer, and lithological contacts are generally gradational. Individual iron–silica bands range from 1.0 to 3.5 mm in thickness and consist of fine- to medium-grained crystals with diameters of ∼0.05–2.0 mm. Magnetite is commonly oxidized to hematite and goethite, while actinolite is partially to completely replaced by goethite. Secondary structures include planar to undulating joints, 2.5–4.0 mm wide, infilled with quartz and sulfide minerals. Later-generation quartz veins, locally reaching thicknesses of up to 5 cm, crosscut earlier joints and microfolds. The BIFs record four deformation events (D1–D4) and four associated episodes of greenschist- to amphibolite-facies metamorphism (M1–M4), expressed by S0–S3 foliations, F1–F3 folding, shear zones, and boudinage structures (Fig. 3b). Foliation is defined by the preferred alignment of muscovite, imparting schistose to locally gneissose textures (Fig. 3c). Deformation features, including fault structures (Fig. 1), are described in detail in Section 2.3. Petrographic analysis of thin sections reveals a mineral assemblage dominated by quartz, hematite, magnetite, muscovite, goethite, actinolite, pyrite, and pyrrhotite.
**Altered massive BIF**	ZDSS02 ZDSS06 ZDRP01 (29.80 m) ZDRP03 (38.80 m) ZDRT01 (38.90 m) ZDRT02 (43.50 m) ZDRT03 (50.10 m)	These samples resemble altered massive to finely laminated BIFs and exhibit slight to moderate weathering textures. They are mechanically competent and resistant to fracture, even when struck with a geological pick. Lithological contacts are generally gradational. The laminae are nearly non-existent, but measuring ∼0.1–0.5 mm in thickness where they occur (Fig. 3). Undulating joints, 2.0–3.5 mm wide, are commonly infilled with quartz crystals. Later-generation quartz veins, with an average thickness of ∼5 cm, are discordant to the earlier joint sets. Microfolds and crenulation structures are observed both in outcrop and in thin section. Isolated boudins of brecciated quartzite occur locally (Fig. 3k). The preferred alignment of muscovite and actinolite defines the schistosity and locally developed gneissose texture. Detailed descriptions of the structural features are provided in Section 2.3. Overall, the ZD Formation BIFs are composed of fine- to medium-grained crystals, with an average grain size of ∼0.5 mm (Fig. 3d, g). Mineralogically, the altered massive BIFs consist predominantly of quartz, magnetite, muscovite, hematite, goethite, and actinolite.
**Least-altered laminated BIF**	ZDRP04 (84.60 m) ZDRP05 (92.30 m) ZDRT04 (106.7 m) ZDRT05 (112.3 m)	This group of samples corresponds to the least-altered laminated BIFs and ranges from unweathered to slightly weathered. The samples are extremely competent and resistant to breakage, even when struck with a geological pick. Lithological boundaries are clear and sharp. Unweathered specimens are dark grey to black (Fig. 3f), whereas weathered equivalents display reddish-brown coloration (Fig. 3h). Laminae thicknesses vary between 1.0 and 2.5 mm (Fig. 3f, h). Planar, smooth to undulating rough joints, measuring 2.0–4.5 mm in width, are infilled with quartz and predate younger quartz veins that range from 2.5 to 5.0 cm in thickness (Fig. 3h). These BIFs comprise fine- to medium-grained crystals with grain sizes between 0.01 and 0.10 mm (Fig. 3m). The preferred orientation of metamorphic minerals defines well-developed schistose to locally gneissose textures. Brecciated chert boudins are a prominent feature in these rocks (Fig. 3c). Petrographic examination of thin sections reveals that the least-altered laminated BIFs are composed predominantly of quartz, magnetite, hematite, goethite, actinolite, muscovite, and subordinate sulfide including pyrite and pyrrhotite.

### 3.3 Geological structures and deformation

Detailed structural measurements collected during fieldwork provide critical constraints on the tectonic architecture, deformation history, and evolutionary development of the ZD Formation. The sequence records multiple deformation events (D1–D4) [14] and currently exhibits a northwest–southeast structural trend with a steep dip of ∼55° to the northeast. Deformation features include folding, faulting, and well-developed shear zones, notably the Houtrivier Shear Zone (HSZ), which extends for more than 100 km and trends northeast ward (Fig. 1). These deformation phases were accompanied by four episodes of regional metamorphism (M1–M4) spanning greenschist to lower amphibolite facies [14,15].

Primary structures related to D1 include F1 folds, thrust faults, and an S1 cleavage developed parallel to primary bedding (S0). The F1 folds are characterized as flat-lying, tight to isoclinal structures, with axial planes subparallel to bedding and foliation. Boundaries between contrasting lithologies, BIF layers, F1 folds, and early shear zones were subsequently overprinted by tight, upright F2 folds that plunge moderately to the southwest. In the field, F2 deformation is expressed as crenulation microfolds displaying Z-asymmetry in planar view and is accompanied by the development of a D2 crenulation cleavage (S2).

The D3 deformation phase is marked by F3 folds expressed as large-scale, upright structures with inclined plunges and a general north–south orientation. The axial planes of the F3 folds are oriented at an angle of ∼10–20° relative to the southwest-plunging F2 folds. The D3 event is further associated with the development of an S3 crenulation cleavage and left-lateral, northeast-striking shear zones. Brittle deformation is manifested by northwest–southeast-trending thrust faults (Fig. 1) that are subvertical and truncate earlier folds, shear zones, faults, and quartz veins. These faults spatially coincide with zones of intense goethite alteration. Overall, foliation defined by S1–S3 cleavages is generally parallel to primary bedding (S0) and steeply plunges toward the northeast at ∼60° (Fig. 1). Foliation is highlighted by the preferred alignment of muscovite, producing schistose to gneissose textures, and is commonly associated with the development of boudin structures (Fig. 3b–c).

Syn-depositional quartz veins that are concordant with BIF banding were deformed concurrently with the host lithologies during early deformation. These concordant veins are subsequently crosscut by later, discordant quartz veins. The concordant veins are typically thin, occurring at millimeter scale, whereas the discordant veins are substantially thicker, ranging from centimeters to meters in length and millimeters in width. Structural relationships indicate that the discordant quartz veins post-date the D1 deformation event but pre-date D3. Quartz veins that transect magnetite-rich layers locally contain disseminated hematite, reflecting post-depositional modification of the iron-rich assemblages [14,15].

### 3.4 Lithological and mineralogical data

**Fig. 2:**
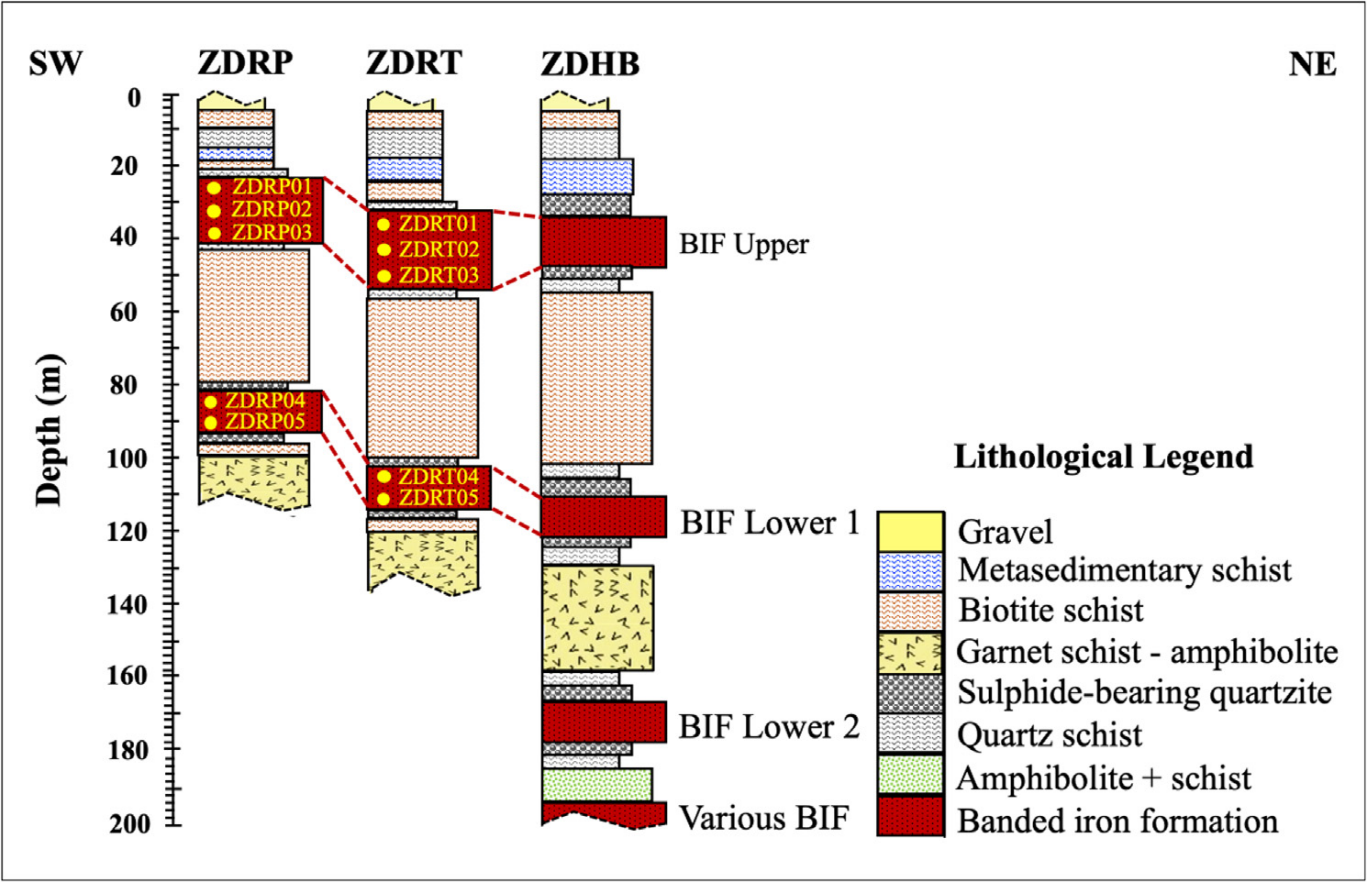
Stratigraphy of Rhenosterkoppies Greenstone Belt created from boreholes ZDRP, ZDRT, and ZDHB [16,17].

The stratigraphy of the ZD Formation consists of BIF orebodies extending northwest–southeast over a distance exceeding 10 km (Fig. 1) and occurring at elevations between 50 and 1400 m above sea level (Fig. 5a–b). Outcropping BIF layers vary in thickness from 3 to 10 m. Field identification of contacts between BIFs and amphibolite is challenging due to intense weathering and slope debris, but these contacts are more clearly observed in drill cores (Fig. 2). Despite these field limitations, the stratigraphic data provide important insights into the distribution, thickness, and structural characteristics of the ZD Formation BIF orebodies.

#### 3.4.1 Amphibolite

**Fig. 3:**
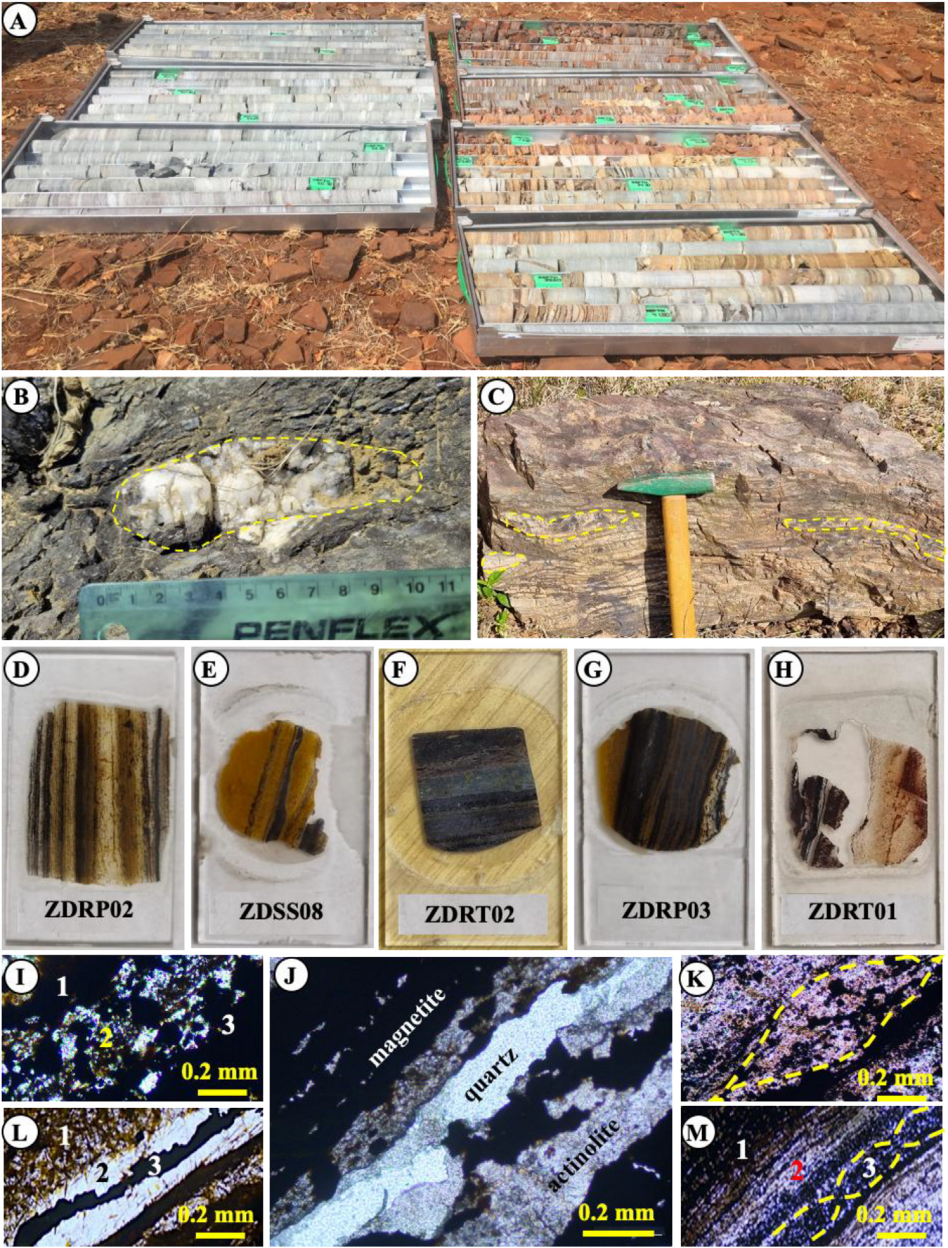
(a) Drill core samples. (b–c) Outcrop BIF samples. (d–h) Thin sections of BIFs displaying typical alternating dark magnetite-bearing layers and bright quartz bands at millimeter scale. (i) Photomicrographs of thin sections showing finely laminated layers: i1 = hematite, i2 = quartz and actinolite partially replaced by ∼10% goethite, i3 = magnetite. (l) l1 = poikilitic actinolite ∼80% replaced by brownish goethite, l2 = quartz, l3 = sulfide minerals (pyrite and chalcopyrite). (j) Massive to finely laminated magnetite, amphibole, and quartz. (k) Boudin structures. (m) m1 = martitized magnetite band, m2 = quartz band, m3 = boudins.

Amphibolite and schist dominate the outcrop, comprising ∼65% of the exposed sequence (Fig. 1). Contacts between amphibolite and other major lithologies strike northwest–southeast and dip 55° northeast. Field observations indicate that primary lithological boundaries have been deformed and transposed toward the main northwest-trending fabric. The amphibolite exhibits a porphyroblastic texture with reddish garnet crystals averaging 3.0 mm in diameter. Laminae within the foliated unit range from 2.5 to 3.5 mm in thickness. Fresh amphibolite is dark grey (Fig. 3a–b), whereas weathered material displays a reddish hue. Primary hornblende is partially to completely replaced by 20% chlorite, actinolite locally replaced by 35% goethite, whereas plagioclase is replaced by 20% clay minerals plus 5% epidote. Quartz occurs as ∼15% boudins and vein fillings, with garnet contributing 5%. Schistosity is well developed, defined by the preferred orientation of hornblende, plagioclase, epidote, biotite, and minor quartz. Banded amphibolite shows distinct quartz-rich boudinage structures (Fig. 3b). Discordant amphibolite dykes, 1–2 m thick, were observed in the field but are localized and poorly exposed.

#### 3.4.2 Schist

The schist is an intermediate-grade metamorphic rock composed of roughly 35% chlorite, 20% quartz, 15% feldspar, 15% biotite, 10% muscovite, and 5% porphyroblastic garnet. Crystal sizes range from fine to medium, averaging ∼2 mm in diameter. The hand specimen’s color varies from greenish due to abundant chlorite to dark grey reflecting the mixture of ferromagnesian minerals with quartz and feldspar. The rock exhibits pervasive schistosity defined by the preferred alignment of chlorite flakes. Schist units are infrequent in the field, typically ranging from 5 to 50 m in thickness following the dominant northwest–southeast structural trend, and are occasionally intercalated within amphibolite sequences.

#### 3.4.3 Quartzite

Quartzite units were observed both in the field and in drill cores, consisting predominantly of ∼95% quartz with ∼5% combined pyrite and pyrrhotite. These units are generally 1–2 m thick and occur either as basement layers or overlying mineralized BIFs, often in close association with schist (Fig. 2). Brecciated quartzite within amphibolite displays pinch-and-swell or boudinage structures (Fig. 3b). Contacts between quartzite and Fe-rich domains are characterized by strong magnetism. The quartz is pure white, and metamorphosed quartz bands, 2–4 cm in diameter, are intercalated within BIFs, forming magnetite-bearing quartzite layers (Fig. 3c).

#### 3.4.4 Gneiss

Gneiss units form the boundary between the upper quartzite and the base of the BIF–amphibolite sequence (Fig. 1). Although these gneisses are well-exposed, field identification of contacts with adjacent units is often obscured by debris from amphibolite and BIF. Fresh exposures revealed well-foliated, coarse-grained gneiss composed of ∼35% K-feldspar, 35% quartz, 25% biotite clusters, and 5% each of amphibole and garnet. Quartz occurs both in light colored bands and veins. The gneissic foliation dips moderately northeast at ∼55°, with strikes trending northwest–southeast (Fig. 1). Gneissic iron-rich bands, observed in the field and thin sections (Fig. 3d–m), exhibit strong magnetic signatures due to the dominance of magnetite over hematite.

#### 3.4.5 Banded iron formations (BIF)

BIF is the second most abundant lithology after mafic amphibolite, accounting for roughly one-quarter of the mapped units. BIF deposits are locally interlayered with alternating resistant lithologies (Fig. 1). Fieldwork identified up to four BIF layers, with ZDRP and ZDRT intersecting two prominent layers, while borehole ZDHB intersects all four (Fig. 1). The BIF sequence includes lower BIF layers, lower BIF 2, lower BIF 1, and upper BIF (Fig. 2). The upper three layers form the main stratigraphy, ranging from 3 to >50 m thick, representing about 90% of the total resource. BIF deposits comprise (a) altered laminated BIF, (b) altered massive to finely laminated BIF, and (c) least-altered laminated BIF.

a) **Altered laminated BIF**
  The altered laminated BIF (Fig. 3) is characterized by alternating 5–10 mm thick layers of dark magnetite or reddish hematite and grey-white quartz, giving the rock a gneissic texture. Magnetite and hematite crystals average ∼1.0 mm, while quartz ranges from 0.05–1.0 mm. Magnetite exhibits stronger magnetic susceptibility, particularly at quartz–chert contacts. Hematite is intergrown with chert/jasper and dolomite (e.g., ZDSS01, ZDSS09; Table 1). Sample ZDSS03 is grey, consisting of 40% hematite, 40% quartz, <10% magnetite, and <5% actinolite and goethite, representing moderately oxidized meta-BIF. Sample ZDRP02 shows laminae 1–2.5 mm thick, with primary magnetite pseudomorphically replaced by martitized hematite (0.1–0.5 mm) martite. Minor magnetite persists as fine inclusions within quartz. Quartz displays slight wavy extinction, and actinolite (0.35 mm) occurs along magnetite–quartz contacts, partially replaced by goethite. Thin goethite veinlets (<0.07 mm) transect laminae parallel or obliquely to layering (Fig. 3l).
b) **Altered massive BIF**
  The altered massive to thinly laminated BIF represents slightly weathered grey meta-BIF (Table 1). Although localized and identified on outcrops, it was not encountered in drilling and constitutes <5% of the total ZD BIF, with thickness <4 m and length <30 m. This unit comprises fine-grained Fe oxides interlayered with graded quartzite. Massive BIF contains minor quartz–chert and grades laterally and vertically into poorly developed thin laminae of BIF, magnetite, and quartz, e.g., ZDRP03 at 38.80 m (Table 1). Quartz is poorly sorted, sub-rounded (∼5 µm), and silica-cemented, rarely preserving primary laminations. Lepidoblastic hematite occurs along lithological contacts, often associated with sheared BIF lenses. The altered-massive BIF comprises 40% quartz, 30% magnetite, 10% muscovite, 5% hematite, and <5% each of goethite and actinolite (Fig. 3j).
c) **Least-altered laminated BIF**
  Least-altered BIF samples include ZDRP04 (84.60 m), ZDRP05 (92.30 m), ZDRT04 (106.7 m), and ZDRT05 (112.3 m) (Table 1), exhibiting fresh, grey colored, layered meta-BIF (Fig. 3f, h). These samples comprise ∼50% quartz, 30% magnetite, 10% actinolite, and <5% each of goethite and hematite. Quartz-rich and Fe oxide-rich bands alternate, ranging from 1.0 to 10 cm in width. Magnetite and actinolite show variable oxidation, with actinolite being more affected (Fig. 3m1). Actinolite occurs as 0.5–2.0 mm poikilitic porphyroblasts embedded within quartz-rich laminae, predominantly associated with magnetite-rich layers, while finer grains (0.2 mm) form dark Fe oxide-dominated laminae up to 0.8 mm thick (Fig. 3d–m). Actinolite is partially replaced by reddish goethite, and magnetite is locally altered to hematite, with crystal sizes of 0.1–0.5 mm. Quartz and magnetite grains are polygonal, granoblastic, and slightly oriented parallel to bedding, with quartz showing minor wavy extinction indicative of tectonic strain.

### 3.5 X-ray diffraction (XRD)

The XRD data show that quartz is the dominant phase across all samples, ranging from ∼31.86 to 50.49 wt.%, indicating silica-rich compositions (Table 3). Magnetite is the principal iron oxide, varying between ∼28.67 and 45.50 wt.%, while hematite varies from ∼3.67 to 21.20 wt.%, reflecting variable oxidation conditions. Iron-rich amphibole occurs in minor but significant amounts, peaking in ZDRT03 (3.91 wt.%), indicating localized alteration influences. Goethite remains relatively consistent (∼1.46–6.10 wt.%), indicating limited secondary weathering. The “Other” category includes muscovite and sulfide minerals (mainly pyrite and chalcopyrite) and generally below 10.00 wt.% except in ZDRT02 (12.17 wt.%), ZDSS06 (10.05 wt.%) depicting compositional anomalies.

**Table 3:** XRD data (wt.%) for 20 selected outcrop and borehole BIF samples. “Other” comprises muscovite, pyrite, and chalcopyrite. MC = magnetic content (%).

Sample ID	Magnetite	Hematite	Quartz	Actinolite	Goethite	Other	Total	MC
ZDSS01	28.90	14.80	40.37	3.35	3.92	5.76	97.10	7.01
ZDSS02	41.60	15.10	40.82	2.10	3.20	2.4	102.82	32.13
ZDSS03	44.75	11.20	34.39	2.00	3.10	6.29	101.73	32.93
ZDSS04	37.80	13.00	35.28	2.20	2.78	9.06	100.12	5.52
ZDSS05	29.30	16.50	40.18	3.21	3.20	7.55	99.94	6.47
ZDSS06	28.55	3.67	50.49	2.84	3.53	10.05	99.13	4.18
ZDSS07	41.94	10.80	31.86	2.00	3.30	8.89	98.79	26.41
ZDSS08	38.20	17.40	40.56	1.89	3.40	2.04	103.49	11.66
ZDSS09	37.50	16.80	41.71	2.20	2.79	2.06	103.06	10.49
ZDSS10	38.91	8.53	37.83	2.10	3.10	9.87	100.34	16.38
ZDRP01	39.55	13.67	40.45	2.84	5.80	1.45	103.76	37.54
ZDRP02	28.67	21.20	35.49	3.01	3.30	8.11	99.78	11.36
ZDRP03	40.50	9.84	36.58	1.94	3.20	4.94	97.00	22.51
ZDRP04	38.89	8.50	45.32	2.03	3.30	4.88	102.92	13.07
ZDRP05	45.50	8.54	40.38	3.03	1.46	1.40	100.31	40.65
ZDRT01	42.40	9.04	38.36	2.20	3.80	7.70	103.50	40.19
ZDRT02	36.50	12.50	35.24	2.10	2.90	12.17	101.41	8.40
ZDRT03	34.01	13.59	40.28	3.91	3.00	6.20	100.99	11.94
ZDRT04	35.80	8.70	42.84	2.78	6.10	2.21	98.43	12.44
ZDRT05	29.30	18.50	42.72	1.96	3.20	6.11	101.79	9.13

### 3.6 Magnetic content

**Fig. 4:**
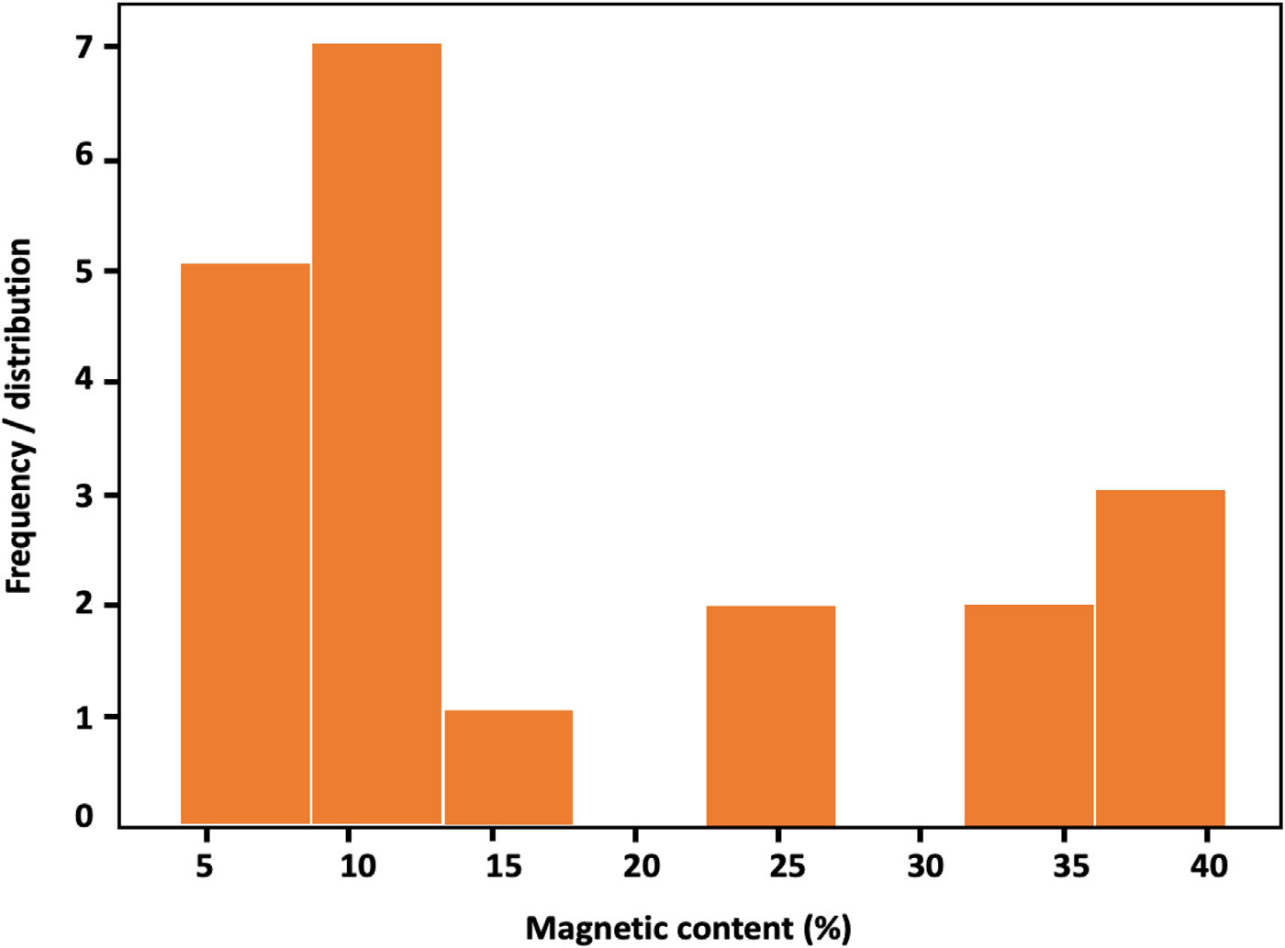
Histogram and distribution plot of Magnetic Content (%), showing a bimodal to slightly right-skewed distribution. The lower mode is primarily associated with outcrop samples, whereas higher magnetic contents correspond to ZDRP and ZDRT borehole samples, indicating distinct compositional populations.

The magnetic content (MC, %) varies from 4.18% (ZDSS06) to 40.65% (ZDRP05), indicating substantial heterogeneity across the data (Table 3, Supporting Materials) [1]. The mean MC is ∼18.00%, indicating a moderate central tendency but with significant dispersion. The data distribution is bimodal to slightly skewed (Fig. 4), with a distinct cluster of lower values largely associated with the outcrop samples.

The lower mode (∼4–12%) corresponds to a relatively low abundance and weaker magnetite and associated with hematite occurrence, particularly in weathered outcrop samples. A few outcrop samples including ZDSS03 (32.93%), ZDSS02 (32.13%), and ZDSS07 (∼26–28%) record comparatively elevated values, indicating least-altered magnetite. Intermediate MC values (∼12–25%) are relatively sparse, creating a separation between populations. Thus, strengthening the bimodal character of the distribution. Overall, the dataset exhibits a slight right skew, driven by a few elevated MC values approaching ∼40%. The higher mode (∼30–41%) is predominantly associated with borehole samples. This grouping reflects significant compositional variability, with certain samples, e.g., ZDRP01 (37.54%), ZDRP05 (40.65%), and ZDRT01 (40.19%) consistently exhibiting elevated MC values. These higher values representing relatively unaltered BIF.

### 3.7 Geochemical data

#### 3.7.1 Major elements geochemistry

Whole-rock geochemical data from 20 selected BIF samples from the ZD Formation indicate a systematic decrease in major and trace oxide abundances in the following order: SiO_2_ > FeO > MgO > CaO > MnO > Al_2_O_3_ > P_2_O_5_ > K_2_O > Na_2_O > BaO > Cr_2_O_3_ > ZrO_2_ > TiO_2_ > SrO > V_2_O_5_ (Table 4). The content of SiO_2_ range from 37.49 to 66.49 wt.% (mean = 42.67 wt.%), whereas total FeO varies between 25.01 and 42.55 wt.% (mean = 39.04 wt.%). The combined MgO, CaO, and MnO contents are consistently low, with average values of 0.71, 0.41, and 0.21 wt.%, respectively. Al_2_O_3_ concentrations range from 0.03 to 0.50 wt.% (mean = 0.18 wt.%). The mean P_2_O_5_ content (0.06 wt.%) is lower than that of Al_2_O_3_ but exceeds the individual concentrations of Na_2_O (0.04–0.10 wt.%) and K_2_O (0.01–0.16 wt.%). Minor oxides, including TiO_2_, V_2_O_5_, BaO, Cr_2_O_3_, SrO, and ZrO_2_, occur at negligible levels in all samples, with concentrations close to the XRF detection limits (Table 4).

**Table 4:** Summary of the descriptive statistics of major elements.

Variables	SiO_2_	FeO	MgO	CaO	Mn_3_O_4_	Al_2_O_3_	P_2_O_5_	K_2_O	Na_2_O	BaO	Cr_2_O_3_	ZrO_2_	TiO_2_	SrO	V_2_O_5_	LOI
**Mean**	42.67	39.04	0.71	0.41	0.21	0.18	0.06	0.08	0.09	0.09	0.10	0.01	0.01	0.01	0.01	0.23
**Standard Error**	1.43	0.86	0.14	0.08	0.02	0.03	0.01	0.01	<0.001	0.01	<0.001	<0.001	<0.001	<0.001	<0.001	0.13
**Median**	41.32	40.05	0.56	0.29	0.21	0.14	0.06	0.10	0.10	0.10	0.10	0.01	0.01	0.01	0.01	0.46
**Mode**	-	-	0.10	0.02	0.17	0.13	0.04	0.10	0.10	0.10	0.10	0.01	0.01	0.01	0.01	0.57
**Std. Deviation**	6.22	3.73	0.61	0.36	0.08	0.13	0.02	0.04	0.02	0.03	<0.001	<0.001	<0.001	<0.001	<0.001	0.57
**Sample Variance**	38.71	13.95	0.38	0.13	0.01	0.02	<0.001	<0.001	<0.001	<0.001	<0.001	<0.001	<0.001	<0.001	<0.001	0.32
**Kurtosis**	13.20	12.03	-0.89	0.53	-0.38	1.62	-0.74	-0.24	3.32	2.55	-2.25	-2.25	-2.25	-2.25	-2.25	-0.85
**Skewness**	3.39	-3.15	0.64	0.91	0.59	1.47	0.67	-0.67	-2.18	-1.90	-1.09	-1.09	-1.09	-1.09	-1.09	-0.69
**Range**	29.00	17.54	1.75	1.30	0.28	0.47	0.07	0.15	0.06	0.09	<0.001	<0.001	<0.001	<0.001	<0.001	1.74
**Minimum**	37.49	25.01	0.09	0.02	0.10	0.03	0.04	0.01	0.04	0.01	0.10	0.01	0.01	0.01	0.01	-0.80
**Maximum**	66.49	42.55	1.84	1.32	0.38	0.50	0.11	0.16	0.10	0.10	0.10	0.01	0.01	0.01	0.01	0.94
**Sum**	810.78	741.76	13.47	7.77	3.96	3.40	1.17	1.55	1.74	1.65	1.90	0.19	0.10	0.10	0.10	4.45
**Count**	20.00	20.00	20.00	20.00	20.00	20.00	20.00	20.00	20.00	20.00	20.00	20.00	20.00	20.00	20.00	20.00
**Conf. level (95%)**	3.00	1.80	0.30	0.17	0.04	0.06	0.01	0.02	0.01	0.01	<0.001	<0.001	<0.001	<0.001	<0.001	0.27

Std. = standard

conf. = confidence

LOI = loss on ignition

(-) = not applicable

SiO_2_ exhibits strong negative correlations with FeO (r = -0.988) and weaker negative correlations with CaO (r = -0.047) and MgO (r = -0.344) (Table 6). Similarly, Al_2_O_3_ shows negative correlations with CaO (r = -0.477), MgO (r = -0.417), and SiO_2_ (r = -0.089) (Table 6). A very weak negative correlation is observed between Al_2_O_3_ and Na_2_O (r = -0.163). In contrast, FeO displays weak positive correlations with CaO (r = 0.047), MgO (r = 0.025), MnO (r = 0.287), and Al_2_O_3_ (r = 0.134). P_2_O_5_ shows a moderate positive correlation with CaO (r = 0.453) and a weak negative correlation with FeO (r = -0.169) (Table 6). Additionally, a very weak negative correlation exists between Al_2_O_3_ and K_2_O (r = -0.175).

**Fig. 5:**
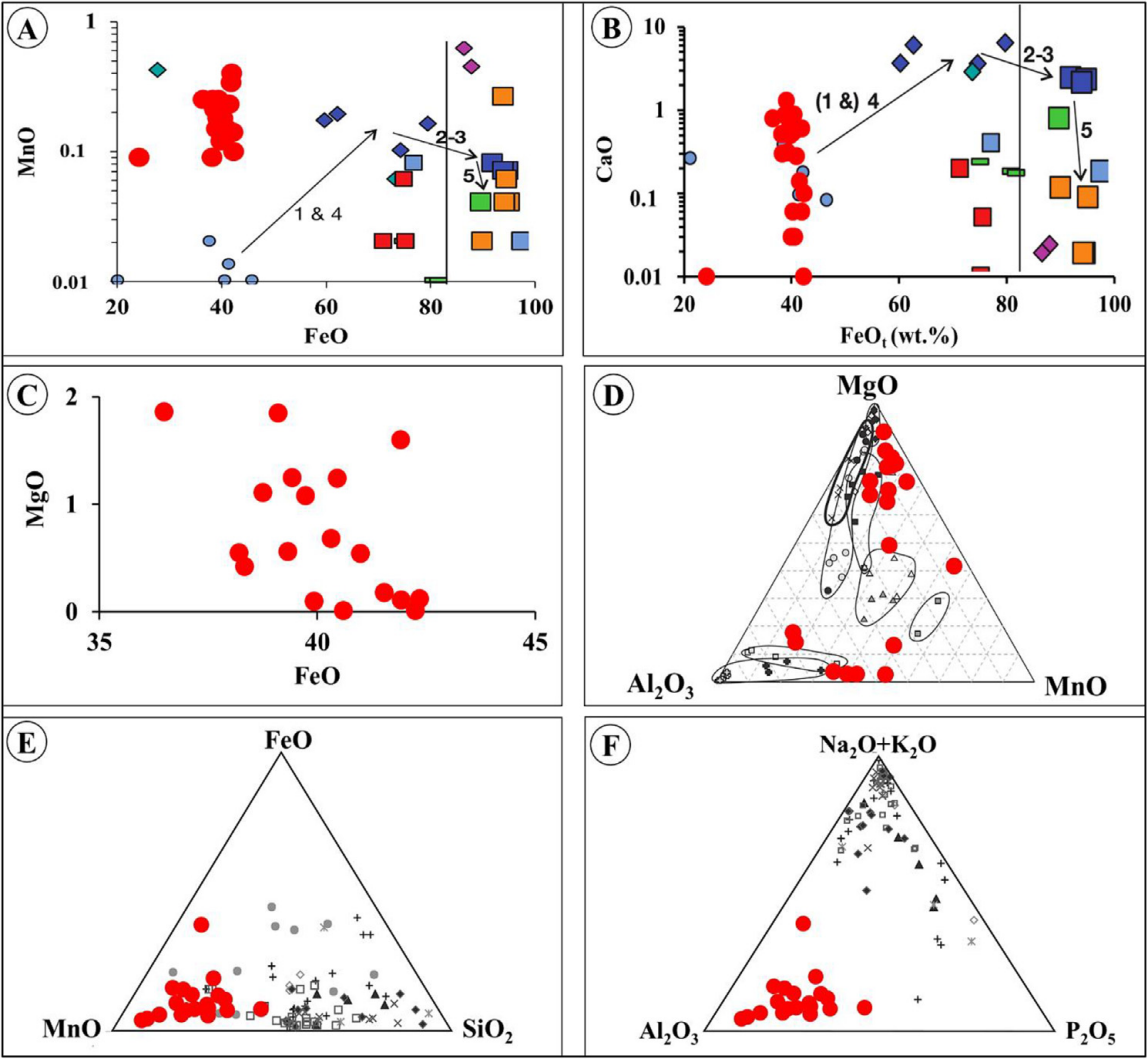
Discrimination diagrams of whole-rock FeO(t) wt.% plotted against selected major oxides wt.% of the least-altered ZD BIF (red dots) in comparison with the Koolyanobbing BIFs, adapted after [12].

Bivariate discrimination diagrams of whole-rock FeO_(t)_ (wt.%) plotted against selected major oxides, including MnO (Fig. 5a), CaO (Fig. 5b), and MgO (Fig. 5c), reveal positive correlations between FeO_(t)_ and each of these oxides. The least altered BIF samples plot predominantly within the Stage 1 and Stage 4 BIF mineralization fields on the FeO_(t)_–MnO and FeO_(t)_–CaO diagrams (Fig. 5a–b) [10]. Ternary plots of MgO + MnO + Al_2_O_3_ indicate that the BIF data are distributed toward the MgO apex, followed by Al_2_O_3_, and away from MnO (Fig. 5d). By contrast, ternary MnO + FeO_(t)_ + SiO_2_ plots show data clustering closer to the MnO apex and away from both FeO_(t)_ and SiO_2_ (Fig. 5e). The ternary diagram of (Na_2_O + K_2_O) + Al_2_O_3_ + P_2_O_5_ shows data distribution toward the Al_2_O_3_ apex (Fig. 5f). Furthermore, the discrimination diagram of (TiO_2_ + V_2_O_5_) versus (Al_2_O_3_ + MnO) places the samples within the hydrothermal temperature range of approximately 200–300°C (Fig. 5c).

#### 3.7.2 Trace elements

**Fig. 6:**
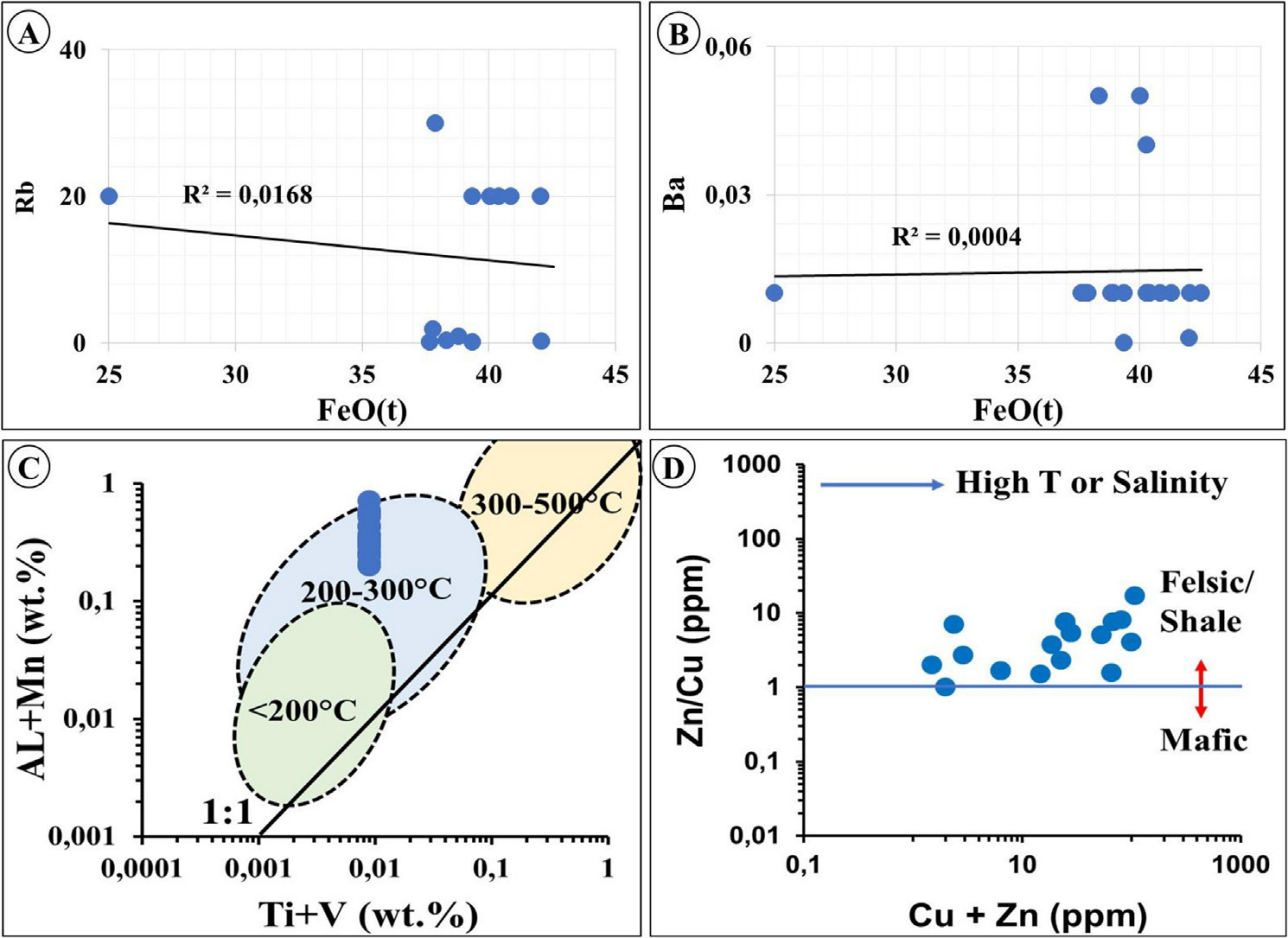
Discrimination diagrams of ZD Fm BIF deposits, adapted after [12].

Trace element concentrations (ppm) of the least altered BIF samples are summarized in Table 5 and in the data repository [1]. The highest concentrations include Ba (520), Co (178.92), and V (132), whereas the lowest values are recorded for U (0.50), Sb (1.29), Cd (1.40), Hg (1.00), and Br (1.00). Although no pronounced overall enrichment is observed, several transition metals, including Ni (0.10–89.60), Cu (0.10–25.89), Pb (0.10–12.00), Ta (0.08–3.10), and W (0.50–3.00), show variable enrichment. The ZD Formation BIFs are depleted in high field strength elements (HFSE), with low mean values of Ta (0.94), Th (0.75), Sc (4.57), Y (32.65), and Zr (8.55). In contrast, large ion lithophile elements (LILE), such as Rb (mean = 11.80) and Sr (mean = 6.02), are relatively elevated, with Ba reaching 480 ppm in sample ZDSS06 and 467 ppm in ZDSS02. A weak negative correlation is observed between FeO_(t)_ and Rb (r = -0.129) (Table 6), whereas FeO_(t)_ shows a very weak positive correlation with Ba (r = 0.267; Fig. 6b). SiO_2_ exhibits weak positive correlations with Zn (r = 0.31) and Zr (r = 0.17) (Table 6). The Zn + Cu versus Zn/Cu discrimination diagram, adapted from [10], places the ZD Formation BIF samples within the felsic or shale sediment source field (Fig. 6d).

**Table 5:** Descriptive statistics summary of trace elements (ppm) of the BIF deposits of the Zandrivierspoort Fm (Supplementary Material). Dashes (-) indicate measurements that are not applicable.

Statistical variables	As	Ba	Cd	Co	Cr	Cs	Cu	Mo	Ni	Pb	Rb	Sb	Sc	Se	Sr	
Mean	4.45	91.98	1.02	9.70	12.39	0.89	7.34	1.40	23.37	3.36	10.80	0.74	4.18	2.27	5.42	
Standard Error	1.03	30.65	0.11	1.61	2.09	0.11	1.49	0.17	5.11	0.87	2.54	0.15	0.75	0.13	1.71	
Median	2.00	51.00	1.00	6.00	16.00	0.86	6.83	1.68	19.00	1.05	6.00	0.40	3.05	2.00	2.81	
Mode	2.00	-	1.00	-	2.00	1.00	-	1.87	-	1.00	0.10	0.31	1.00	2.00	0.70	
Standard Deviation	4.47	133.58	0.49	7.02	9.12	0.48	6.48	0.75	22.27	3.78	11.08	0.67	3.27	0.56	7.45	
Sample Variance	19.96	17843.86	0.24	49.34	83.10	0.23	42.04	0.56	495.97	14.31	122.80	0.45	10.70	0.32	55.48	
Kurtosis	0.29	5.34	0.83	-1.73	-1.74	2.70	2.96	-1.63	3.25	1.24	-1.43	4.78	0.22	1.28	5.32	
Skewness	1.39	2.48	0.56	0.31	-0.03	1.42	1.61	-0.26	1.60	1.36	0.41	2.15	0.99	0.75	2.30	
Range	13.00	474.74	1.98	19.93	25.45	2.03	25.59	2.05	88.50	12.96	31.90	2.54	11.17	2.53	28.18	
Minimum	1.00	1.39	0.10	1.07	1.00	0.09	0.30	0.29	1.10	0.10	0.10	0.28	0.88	1.17	0.60	
Maximum	14.00	476.13	2.08	21.00	26.45	2.12	25.89	2.34	89.60	13.06	32.00	2.82	12.05	3.70	28.78	
Sum	84.59	1747.61	19.33	184.34	235.37	16.87	139.40	26.64	444.04	63.79	205.20	14.15	79.39	43.05	103.06	
Count	20.00	20.00	20.00	20.00	20.00	20.00	20.00	20.00	20.00	20.00	20.00	20.00	20.00	20.00	20.00	
Confidence 95%	2.15	64.38	0.23	3.39	4.39	0.23	3.12	0.36	10.73	1.82	5.34	0.32	1.58	0.27	3.59	
**Statistical variables**	**Ta**	**Th**	**U**	**V**	**W**	**Zn**	**Zr**	**La**	**Ce**	**Nd**	**Sm**	**Eu**	**Tb**	**Y**	**Yb**	**Lu**
Mean	1.64	1.38	3.41	26.60	3.85	29.36	9.56	10.90	11.40	9.03	2.04	0.74	0.27	6.25	0.69	0.10
Standard Error	0.56	0.17	2.65	7.04	0.80	7.11	3.09	2.88	3.09	2.70	0.60	0.20	0.07	1.15	0.14	0.02
Median	1.05	1.08	0.68	14.00	2.71	20.95	2.88	2.86	4.09	1.99	0.43	0.27	0.12	4.46	0.56	0.07
Mode	1.00	2.00	0.69	9.00	2.94	2.10	1.00	-	-	-	-	0.65	0.07	-	-	0.06
Standard Deviation	2.43	0.73	11.54	30.67	3.48	30.97	13.47	12.55	13.47	11.75	2.60	0.86	0.30	5.01	0.59	0.08
Sample Variance	5.90	0.53	133.19	940.35	12.11	959.15	181.40	157.47	181.41	138.11	6.78	0.73	0.09	25.10	0.35	0.01
Kurtosis	16.31	-1.47	18.87	6.62	13.92	0.65	3.70	-0.67	0.72	2.04	1.76	0.17	-0.02	1.39	0.66	1.77
Skewness	3.91	-0.16	4.34	2.41	3.57	1.25	1.95	0.97	1.39	1.60	1.54	1.33	1.21	1.31	1.21	1.46
Range	11.27	2.27	50.80	123.74	15.41	100.13	49.50	34.97	43.78	41.05	8.84	2.48	0.89	18.43	1.97	0.30
Minimum	0.08	0.13	0.20	5.60	1.91	1.00	0.50	0.72	1.12	0.96	0.20	0.13	0.04	0.53	0.04	0.01
Maximum	11.35	2.40	51.00	129.34	17.32	101.13	50.00	35.69	44.90	42.01	9.04	2.61	0.93	18.96	2.01	0.31
Sum	31.13	26.17	64.81	505.42	73.12	557.84	181.69	207.19	216.60	171.54	38.81	14.05	5.21	118.79	13.20	1.95
Count	20.00	20.00	20.00	20.00	20.00	20.00	20.00	20.00	20.00	20.00	20.00	20.00	20.00	20.00	20.00	20.00
Confidence 95%	1.17	0.35	5.56	14.78	1.68	14.93	6.49	6.05	6.49	5.66	1.26	0.41	0.14	2.41	0.29	0.04

**Table 6:** Pearson correlation matrix of geochemical data. The correlation coefficient ranges between (+1) denoting strong positive relationship shown in deep blue and (-1) mirroring strong negative correlation highlighted by deep red [1].

wt.% = weight percentage: major elements

ppm = parts per million: trace elements

MC = Magnetic Content

LOI = loss on ignition

#### 3.7.3 Rare earth elements

The average rare earth element (REE) concentrations (ppm) in the ZD Formation BIF samples decrease in the following order: Ce (11.13) > La (10.42) > Nd (8.57) > Y (6.26) > Sm (1.91) > Yb (0.66) > Eu (0.61) > Tb (0.26) > Lu (0.09). Total REE contents (ΣREE) range from 6.77 to 111.88 ppm, with a mean value of 39.90 ppm. Pearson’s correlation matrix (Table 6) and binary plots (Fig. 6) show that FeO_(t)_ is negatively correlated with individual REEs, including La (r = -0.420), Ce (r = -0.219), Nd (r = -0.549), Sm (r = -0.584), Eu (r = -0.420), Tb (r = -0.510), Y (r = -0.546), Yb (r = -0.438), and Lu (r = -0.401). In contrast, SiO_2_ exhibits positive correlations with Sm (r = 0.55), Y (r = 0.55), Nd (r = 0.54), Tb (r = 0.54), Eu (r = 0.43), Yb (r = 0.44), La (r = 0.39), Lu (r = 0.39), and Ce (r = 0.21) (Table 6). Positive inter-element correlations among the REEs are also observed (Table 6).

Preferential enrichment of light rare earth elements (LREE) relative to heavy rare earth elements (HREE) is indicated by chondrite-normalized ratios of (La/Sm) (Supplementary Material). The chondrite-normalized REE patterns display pronounced LREE fractionation, with a mean (La/Yb) ratio of 18.10. Enrichment of LREE is further reflected by an average (La/Sm) value of 3.52, compared with a lower HREE ratio, (Tb/Yb) = 2.75. The ZD Formation BIF samples show a weak positive Eu anomaly, with Eu/Eu* averaging 0.08 under chondrite normalization [12,18]. Total REE contents (ΣREE) vary widely, ranging from 6.77 ppm in sample ZDSS10, which exhibits the weakest Eu anomaly, to 111.88 ppm in sample ZDSS02. Sample ZDSS05 records an intermediate ΣREE value of 65.83 ppm and is notable for its relatively elevated Al_2_O_3_ content (0.50 wt.%). In addition, the REE patterns show a weak negative Ce anomaly, with Ce/Ce* = -0.82 (Table 5).

Exploration drilling data from boreholes ZDRP, ZDRT, and ZDHB, together with historical drill core data from Anglo American, were statistically analyzed to evaluate Fe grades (%) within the meta-BIF deposits over a cumulative BIF thickness of approximately 40 m. Iron grades are consistently distributed across the area investigated and correspond closely with assay data from more than 50 historical exploration drill cores, which report Fe grades ranging from 32 to 40%. Based on the present dataset and supplemented by Anglo American’s 2017 resource statement, the ZD Formation Fe ore deposits comprise measured and indicated resources totalling 313.41 Mt. Of this, approximately 42% Fe is hosted in magnetite (Fe_3_O_4_) at a cut-off grade of ∼22% Fe. The inferred resource is estimated at 163 Mt at an average grade of 36% Fe (Fe_3_O_4_). Accordingly, the total mineral resource for the ZD Formation Fe ores is estimated at approximately 475 Mt, with an average grade of 35.5% Fe and an average Fe_3_O_4_ grade of 40% Fe [17].

## 4 Experimental Design, Materials and Methods

### 4.1 Field mapping and data collection

Detailed non-invasive fieldwork was conducted at a scale of 1:10,000 to undertake geological and structural mapping over an area of approximately 16 km^2^, centred at latitude 23°37′17.21″ and longitude 29°38′59.99″ (Fig. 1). The area under investigation was selected due to excellent exposure of the ZD Formation stratigraphic sequence, which hosts BIF orebodies extending for ca. 8 km along strike and up to 2 km in width. Geologically, the ZD Formation comprises a series of strata-bound lenses associated with BIF units, exposed as prominent, steeply dipping ridges. Several lithological units were identified in outcrop, including BIF, quartzite, schist, gneiss, and amphibolite.

Lithofacies containing variable proportions of magnetite and hematite within the ZD Formation BIF deposits were identified and classified as hard or soft, weathered or fresh, and altered or least altered (Table 2). Outcropping BIF thicknesses, measured using a 30 m measuring tape, range from 3 to 10 m, with an average thickness of approximately 7 m. Outcropping lithological contacts between BIF units and adjacent lithologies were difficult to observe in the field owing to extensive weathering and talus cover along slopes; however, these contacts were delineated using drill core information.

**Table 2:** Geological logging sheet for borehole ZDRP.

Rhenosterkoppies Greenstone Belt (RGB) Sequence																	
Depth (m)		Rock Type	W	H	Run Length	TCR	SCR	RQD	Rubble zone		Joint Condition						
From	To								From	To	From	To	Angle	Th	#	R	Alteration
0.00	0.85	GRV	W5	H2	0.85	0.30	0.10	0.00	-	-	-	-	-	-	-	-	-
0.85	2.80	DOL	W5	H2	1.95	0.35	0.10	0.00	-	-	-	-	-	-	-	-	-
2.80	6.50	DOL	W5	H2	3.70	0.35	0.00	0.00	-	-	-	-	-	-	-	-	-
6.50	7.35	SCB	W4	H2	0.85	0.25	0.00	0.00	-	-	-	-	-	-	-	-	-
7.35	8.65	SCQ	W3	H2	1.30	1.25	0.45	0.00	7.65	8.90	7.30	7.50	65	2	-	2	1
8.65	9.80	SCQ	W3	H2	1.15	0.70	0.20	0.00	8.90	10.35	-	-	-	-	-	-	-
9.80	12.00	SCQ	W3	H2	2.20	1.50	0.40	0.00	10.35	11.50	11.40	11.90	65	1	-	2	1
12.00	13.20	SCQ	W3	H2	1.20	1.10	0.30	0.00	12.50	13.25	12.00	12.50	60	2	-	2	1
13.20	14.50	SCQ	W3	H2	1.30	1.15	0.40	0.00	13.25	14.50	13.75	14.20	15	1	-	5	1
14.50	15.95	SCQ	W3	H2	1.45	1.30	1.05	21.20	16.05	16.20	14.65	16.05	70	1	15	2	1
15.95	17.90	SCM	W3	H2	1.95	1.30	0.60	0.00	17.45	17.90	16.20	17.45	70	1	23	2	1
17.90	20.00	SCM	W3	H2	2.10	1.85	1.70	5.55	18.40	19.25	17.90	20.05	60	2	28	5	1
20.00	20.90	SCM	W3	H2	0.90	0.50	0.10	0.00	20.05	20.90	-	-	-	-	-	-	-
20.90	23.80	SCB	W4	H2	2.90	1.95	1.80	50.00	21.35	23.80	21.65	23.90	60	2	11	6	-
23.80	25.25	SCQ	W4	H2	1.45	2.00	0.70	0.00	23.80	25.20	-	-	-	-	-	-	-
25.25	26.80	SCB	W4	H3	1.55	0.55	0.05	0.00	25.20	26.80	-	-	-	-	-	-	-
26.80	29.80	SCB	W5	H4	3.00	2.10	1.80	22.30	29.20	29.40	29.45	29.80	60	3	27	2	-
29.80	33.00	BIS	W2	H5	3.20	1.45	0.90	0.00	29.80	29.95	29.95	32.75	70	1	20	6	-
33.00	34.15	BIS	W2	H5	1.15	0.95	0.80	16.15	33.05	33.50	33.50	34.10	60	2	15	9	-
34.15	35.25	BIS	W2	H5	1.10	1.00	0.80	51.70	-	-	34.25	35.35	70	2	12	6	-
35.25	35.80	BIS	W2	H5	0.55	0.55	0.15	0.00	-	-	35.25	35.80	70	2	9	6	-
35.80	38.00	BIS	W2	H5	2.20	1.70	1.35	21.85	-	-	35.85	37.90	75	1	23	2	-
38.00	39.55	BIS	W2	H5	1.55	1.40	0.45	7.35	39.00	39.55	37.90	39.60	75	2	21	2	-
39.55	41.40	BIS	W2	H5	1.85	1.50	0.20	0.00	39.55	41.40	-	-	-	-	-	-	-
41.40	42.40	BIS	W2	H5	1.00	0.70	0.45	24.00	41.45	41.80	41.85	42.30	70	1	4	5	-
42.40	43.45	BIS	W2	H5	1.05	0.85	0.35	17.90	42.55	42.70	42.40	43.45	45	2	5	2	-
43.45	46.30	BIS	W2	H5	2.85	2.65	1.70	44.20	44.45	44.75	43.45	46.10	20	2	12	3	2
46.30	48.60	BIS	W2	H5	2.30	0.45	0.00	0.00	46.30	48.60	-	-	-	-	-	-	-
48.60	50.30	BIS	W2	H5	1.70	1.40	0.70	14.30	48.60	48.95	48.85	50.30	50	2	10	6	2
50.30	52.00	BIS	W2	H5	1.70	1.65	1.15	47.25	-	-	50.50	51.95	75	2	13	6	2
52.00	55.30	SCQ	W3	H3	3.30	2.95	2.20	13.70	-	-	51.95	55.30	70	2	41	3	2
55.30	57.00	SCB	W3	H3	1.70	1.25	0.80	41.55	-	-	55.50	56.80	75	2	11	2	2
57.00	58.30	SCB	W3	H3	1.30	1.45	1.35	92.55	-	-	57.55	57.95	75	2	5	2	2
58.30	59.65	SCB	W3	H3	1.35	1.30	1.20	69.85	-	-	58.35	59.40	75	1	7	2	2
59.65	62.80	SCB	W3	H3	3.15	2.90	2.80	59.25	60.40	60.70	59.75	61.30	80	3	14	6	2
62.80	64.20	SCB	W3	H3	1.40	1.30	1.25	67.15	64.05	64.15	63.05	63.95	75	1	6	5	2
64.20	65.20	SCB	W3	H3	1.00	1.00	0.95	93.25	-	-	64.40	64.95	80	2	4	5	2
65.20	66.15	SCB	W3	H3	0.95	0.95	0.90	87.90	-	-	66.05	66.15	75	2	2	6	2
66.15	68.80	SCB	W3	H3	2.65	2.70	2.70	83.00	-	-	66.25	68.60	70	2	13	9	2
68.80	71.80	SCB	W3	H3	3.00	2.95	2.90	94.30	-	-	69.25	71.60	75	3	10	6	2
71.80	73.65	SCB	W3	H3	1.85	1.65	1.55	72.95	-	-	71.95	73.35	75	2	7	5	2
73.65	76.20	SCB	W3	H3	2.55	2.70	2.40	93.00	-	-	73.70	76.25	80	1	8	2	2
76.20	78.10	SCB	W3	H3	1.90	1.95	1.60	73.70	-	-	76.20	78.15	80	2	9	6	2
78.10	79.50	SCB	W3	H3	1.40	1.35	1.25	92.05	-	-	78.45	79.50	83	2	7	6	2
79.50	82.00	SCB	W3	H3	2.50	2.50	2.50	84.95	-	-	79.80	81.85	80	2	12	2	2
82.00	84.00	SCB	W3	H3	2.00	1.80	1.65	82.15	-	-	82.70	83.90	88	2	10	2	2
84.00	86.00	SCB	W3	H3	2.00	2.95	2.65	53.40	-	-	89.90	86.80	70	2	26	2	2
86.00	90.00	SCB	W3	H3	4.00	3.05	2.75	76.65	89.70	89.80	87.15	89.70	75	2	15	8	2
90.00	91.50	SCB	W3	H3	1.50	1.95	1.06	46.40	90.90	91.75	89.80	91.75	70	2	18	6	2
91.50	93.40	SCB	W3	H3	1.90	1.75	1.10	27.90	91.95	92.50	92.60	93.30	60	2	7	9	2
93.40	95.20	SCB	W3	H3	1.80	1.70	1.60	82.30	-	-	93.55	95.70	80	2	9	5	2
95.20	95.70	SCB	W3	H3	0.50	0.60	0.60	117.00	-	-	95.20	95.75	60	2	4	3	2
95.70	97.30	SCB	W3	H3	1.60	1.40	1.40	61.80	-	-	95.90	97.20	80	2	9	8	2
97.30	97.85	SCB	W3	H3	0.55	0.50	0.40	27.30	-	-	97.45	97.80	85	2	9	6	2
97.85	100.30	PBR	W2	H4	2.45	2.30	2.30	89.40	-	-	98.10	5.00	75	2	7	2	2
100.30	103.30	BIS	W2	H4	3.00	3.00	2.95	84.00	-	-	100.45	103.3	80	2	14	2	2
103.30	105.10	BIS	W2	H4	1.80	1.85	1.50	45.55	104.45	104.65	103.50	104.9	75	2	14	2	2
105.10	107.80	BIS	W1	H4	2.70	2.70	2.25	54.80	107.00	107.10	105.50	107.5	75	2	18	2	2
107.80	110.80	BIS	W1	H4	3.00	2.90	2.90	80.35	-	-	107.90	110.8	80	2	12	8	2
110.80	111.85	PBR	W1	H4	1.05	1.15	0.70	67.60	111.35	111.85	111.15	111.4	70	2	4	5	2
111.85	114.30	SCB	W1	H4	2.45	2.40	2.30	84.10	-	-	111.85	114.3	80	2	11	6	2
114.30	115.00	SCB	W1	H4	0.70	0.75	0.70	73.20	-	-	114.50	114.9	80	2	3	6	2
115.00	118.30	SCB	W1	H4	3.30	30.4	2.80	69.20	115.90	116.00	116.10	118.3	75	2	14	9	2
118.30	121.30	SCB	W1	H4	3.00	2.85	2.75	81.65	-	-	118.30	121.2	85	2	11	2	2
121.30	124.30	SCB	W1	H4	3.00	3.05	3.05	98.35	-	-	121.85	124.2	80	2	7	5	2
124.30	127.30	SCG	W1	H4	3.00	2.95	2.90	86.00	-	-	124.85	127.0	85	2	6	2	2
127.30	130.30	SCG	W1	H4	3.00	3.05	3.10	93.65	-	-	127.40	128.9	70	2	9	9	2
130.30	135.00	SCB	W1	H4	4.70	2.70	2.65	87.65	-	-	13155	13190	75	2	2	6	2

**Hardness**				**Weathering Status**						**R = Joint Roughness**				**Vein Types**			

H1	Very Soft			W1	Unweathered					1	Planar slickensides			1	Qtz = Quartz		
H2	Soft			W2	Slightly weathered					2	Planar smooth			2	Cal = Calcite		
H3	Hard			W3	Moderately weathered					3	Planar rough			3	Hm = Hematite		
H4	Very Hard			W4	Highly weathered					4	Undulating slickensides			4	Mt = Magnetite		
H5	Extremely Hard			W5	Completely weathered					5	Undulating smooth			5	Chl = Chlorite		
										6	Undulating rough			6	Ep = Epidote		
TCR = Total Core Run										7	Stepped slickensides			7	Spd = Sulfide		
SCR = Solid Core Run										8	Stepped smooth						
RQD = Rock Quality Designation										9	Stepped rough						
GRV				Gravel /Talus						**Joint Conditions**							
SCM				Metasedimentary schist (Si, K, Al)						1				No alteration			
SCB				Biotite schist (Fe, Mg, K, Al, rich)						2				Weaker than the IRS			
SCG				Garnet, biotite bearing schist (Fe, Mg, Chl, Qtz)						3				Stronger than the IRS			
SCQ				Quartzitic schist (Si-rich)													
AMPH				Amphibole, amphibolitic schist (Fe, Mg)						**Th = Joint Thickness**							
PBR				Pyrrhotite breccia						1				< 1 mm			
BIS				Banded iron stone, Itabirite (meta-BIF)						2				1 - 5 mm			
DOR				Dolerite						3				> 5 mm			

Dashes (-) means no data measurements.

Structural data, including measurements of bedding planes, folds, faults, and foliation, were collected using a Brunton-type silver compass, except in magnetic BIF layers where a clinometer ruler was utilized. Magnetic susceptibility and relative magnetic strength of the BIF units were measured using a magnetic pen. A lithological map (Fig. 1) and borehole correction diagram (Fig. 2) were generated from field data. The resulting geological map illustrates the distribution of local lithologies and associated BIF mineralization zones. Relatively fresh samples for mineralogical and geochemical data were obtained by breaking weathered surfaces with a geological hammer.

### 4.2 Core drilling and geological core logging

Limited surface exposure hindered effective geological field mapping; however, historical exploration boreholes ZDRT, ZDRP, and ZDHB drilled by Anglo American were re-examined in 2020 and utilized for geological data acquisition. Drill core data from these boreholes were used to construct subsurface lithological logs (Fig. 2). Although the precise geographic coordinates of individual boreholes are not disclosed due to company confidentiality, their relative positions are indicated on the compiled geological map (Fig. 1).

The drilling programme employed reverse circulation (RC) percussion using 50 mm diameter diamond drill bits (Fig. 3a). Boreholes were inclined and drilled approximately perpendicular to bedding planes and geological contacts, reaching maximum depths of 115 m (ZDRP), 135 m (ZDRT), and approximately 200 m (ZDHB) (Fig. 2; Table 2). Boreholes ZDRP and ZDRT were selected for detailed analysis based on their broad spatial distribution, sufficient depth to penetrate below the near-surface weathering profile, and their potential to intersect multiple targeted BIF units.

Geological core logging was conducted in September 2020, focusing on boreholes ZDRP and ZDRT. A total of 240 m of drill core, stored in 12 core trays and extending from surface to below ground level (bgl), were logged. The borehole data provided detailed information on subsurface lithologies, thicknesses, chemical composition, mineralization style and form, geological structures, textures, and degrees of weathering and alteration, with particular emphasis on BIF horizons (Table 2). The principal rock types identified include pyrrhotite-bearing quartzite, amphibolite, BIF, and schist at various stratigraphic levels (Fig. 2). These geological data were subsequently used to estimate Fe ore volumes and grades for the ZD Formation BIF deposits.

### 4.3 Sample collection

A total of 20 representative samples of the ZD Formation BIFs were collected from both surface outcrops and drill cores to capture the variability of iron mineralization. Ten drill core samples were obtained from boreholes ZDRP and ZDRT, whereas additional ten samples were collected from exposed outcrops (Table 1). Drill core depths were recorded and used to construct lithological logs to document stratigraphic relationships (Fig. 2). Following completion of geological core logging, five BIF samples were collected from each borehole. The locations of surface samples were determined using a Global Positioning System (GPS) and are shown on the geological map (Fig. 1).

Sample selection was designed to capture variability in BIF characteristics and to ensure representative coverage of *in situ* Fe mineralization across the mapped area. Drill cores were aligned and longitudinally split into axial halves prior to sampling, with fresh, least altered, and unweathered BIF intervals preferentially targeted at selected depths. One half of each split core was returned to the core trays and retained in core storage, while the corresponding halves, weighing approximately 250–500 g, were placed in plastic sample bags, labelled with unique sample identifiers. These samples were dispatched to accredited laboratories for preparation of samples for analysis using a combination of petrological (Fig. 3a–h), mineralogical (Fig. 3i–m), and whole-rock geochemical techniques (Tables 3,4).

### 4.4 Petrography

The BIF samples were cut into small blocks using an electric rock saw for thin-section preparation. The specimens were subsequently polished to standard thin sections with a thickness of ∼0.30 mm (Fig. 3d–h). Representative subsamples of the selected BIFs were examined for microstructural and mineralogical characteristics using ore microscopy. Thin section analysis was performed using both transmitted- and reflected-light microscopy. Microphotographs were captured with a 3 MP (2048 × 1536) CMOS camera attached to the microscope (Fig. 3d–h).

### 4.5 X-ray diffraction (XRD)

Quantitative X-ray diffraction (XRD) analysis was performed on the pulverized aliquot. Each powdered sample was uniformly distributed on a circular stainless-steel sample holder, levelled using a glass slide, and covered with Kapton film. The pulverized samples were micronized in ethanol using a McCrone micronizer for 10 minutes, after which they were centrifuged to remove excess ethanol and subsequently dried at 60°C. XRD data were collected over a range of 5–140° 2θ using a PANalytical MPD diffractometer equipped with a cobalt tube (Co Kα radiation), operated at 40 kV and 40 mA.

Representative aliquots were pulverized and analyzed by XRD to identify the major crystalline phases present (>1-3%). XRD were measured using a PANalytical diffractometer that employed co-radiation. Quantitative phase analysis was performed using PANalytical HighScore Plus software package and the ICSD database [19]. Crystal structure models used in the refinement included magnetite (Fe_3_O_4_), hematite (Fe_2_O_3_), goethite (FeO.(OH)), Fe-rich Amphibole ((Ca,Fe)_2_Fe_5_Si_8_O_22_(OH)_2_), and Quartz (SiO_2_). Mineral quantities were estimated using the Rietveld Refinement method and the data are given in Table 3 and used for setting up saturation magnetic analysis (SATMAGAN) procedure (Section 4.6).

### 4.6 Saturation magnetic analysis (SATMAGAN)

Magnetic fractions of BIF samples were attained using magnetic separation after 48-hour sun-drying to prevent overheating of the powder samples. Samples were crushed to ∼95% passing 3.0 mm using a jaw crusher. Each sample was homogenized and subjected to a 3-point milling curve to determine the optimal milling time in a swing-disk mill, targeting ∼95% passing 150 µm particle size. Approximately 20 g aliquots were analyzed at SGS, Randburg, South Africa using a saturation magnetic analysis (SATMAGAN) 135 [20]. The experimental parameters were set up with a limit of detection of 0.1 wt.% at the temperature of 10-40°C, humidity of <95% and at a measuring time of 60 seconds per sample.

Roasted, pulverized samples were placed in 1.2 ml acrylic containers and analyzed for saturation magnetism. Triplicate measurements yielded a standard deviation of 0.01, with demagnetization between runs to ensure accuracy and consistency of the readings. Higher SATMAGAN values indicate increased magnetite content (Table 3). The SATMAGAN readings were measured against quantitative XRD magnetite wt.% using representative samples (Table 3). A linear relationship with ±0.02 error was established between s SATMAGAN values and magnetite content.

### 4.7 X-ray fluorescence (XRF)

Major-element geochemical data were generated in Randburg, South Africa, using BIF samples analyzed by X-ray fluorescence (XRF) (Table 4). Samples were air-dried at room temperature for two days prior to analysis. The dried material was crushed using a steel jaw crusher to a nominal particle size of approximately 3.0 mm. Each sample was thoroughly homogenized and split into two aliquots, one designated for major-element analysis and the other for trace-element determination. Approximately 200 g of material from each sample, obtained using a riffle splitter, was milled to <100 µm using a tungsten–carbide swing-disk mill. To minimize potential cross contamination between samples, the milling pot was periodically cleaned with quartz grains before processing subsequent samples. Milling durations ranged from 2 to 4 min, after which the material was sieved to achieve a target of ∼95% passing 150 µm. Powdered samples were pressed into pellets under a compressive pressure of 50 bar for 2–3 min and subsequently oven-dried at 60°C for 120 min.

After cooling, the reduction in sample mass was measured to determine loss on ignition (LOI). The accuracy of major-oxide data for the BIF standard falls within the typical uncertainty of XRF measurements, with detection limits and analytical precision (2σ standard deviation) calibrated at 0.005 wt.% for major oxides. Approximately 55 g of milled samples were pressed into a powder briquette and analyzed for15 major oxides using a PANalytical Axios X-ray fluorescence spectrometer equipped with a 4 kW Rh tube. The analysis was performed at SANS accredited UIS Analytical Service in Centurion, Gauteng, South Africa. Raw concentrations of major elements and oxides, including FeO, SiO_2_, Al_2_O_3_, K_2_O, P_2_O_5_, Mn_3_O_4_, CaO, MgO, TiO_2_, Na_2_O, V_2_O_5_, BaO, Cr_2_O_3_, SrO, ZrO_2_ (wt.%), are summarized in Table 4 and provided in the online Supplementary Material hosted in the Mendeley Data repository [1]. Most oxides show analytical errors of <0.15%. Loss on ignition was calculated after heating samples to temperatures exceeding 650°C.

### 4.8 LA-ICP-MS

Trace elements and REE concentrations were determined using inductively coupled plasma mass spectrometry (ICP–MS), complemented by instrumental neutron activation analysis (INAA), to characterize the geochemistry of the BIF samples. Portions of the samples previously crushed and milled using a mild-steel vibrating disc pulverizer at SGS Laboratories were used for whole-rock trace elements and REE data. Approximately 5 g of powdered material was prepared by fusion in graphite crucibles at 950°C prior to analysis. Trace-element and REE concentrations are reported in parts per million (ppm), with detection limits generally ranging from 0.1 to 1.0 ppm; vanadium represents an exception, with a detection limit of 8.0 ppm. Analytical precision is reported at the 2σ level.

The accuracy of the REE data falls within the typical uncertainty of ICP–MS data, and analytical precision is within 15% of the measured concentrations. Rare earth element concentrations were normalized to Ivuna-type carbonaceous chondrite (CI) values by multiplying the reference values by a factor of 1.36, following the normalization procedures of [2,3]. Eu and Ce anomalies were evaluated using Eu/Eu* = Eu_n_/(0.67Sm_n_ + 0.33Tb_n_) and Ce/Ce* = log[Ce_n_/(La_n_ x Nd_n_)^1/2^] [[4], [5], [6]].

## Limitations

The main limitation relates to constraints in funding, which restricted the number of samples that could be collected and analyzed. The dataset volume is relatively limited and may not fully capture the spatial variability of the BIFs and associated iron ore mineralization across the PGB. This limitation also affected the robustness of statistical analysis and the representativeness of geochemical trends. Future datasets incorporating a larger sample set and additional borehole data will improve statistical reliability and regional interpretation.

## Ethics Statement

The authors have read and followed the ethical requirements for publication in Data in Brief and confirming that the current work does not involve human subjects, animal experiments, or any data collected from social media platforms.

## CRediT Author Statement

**Lowanika Victor Tibane:** Conceptualization, Project administration, Funding acquisition, Methodology, Data curation, Writing- Original draft preparation, Visualization, Investigation, Data validation, Writing- Reviewing and Editing; **David Mamba:** Conceptualization, Project administration, Methodology, Visualization, Investigation, Data validation, Writing- Reviewing and Editing; **Zakhele Handsome Nkosi:** Methodology, Data curation, Visualization, Investigation, Data validation, Writing- Reviewing and Editing.

## Data Availability

Mendeley DataGeochemical Data of Major and Trace Elements of the BIF of ZD Fm of the RGB (Original data).

## Appendix Supplementary materials

Image, application 1
